# Noncontact Strain Monitoring of Osseointegrated Prostheses

**DOI:** 10.3390/s18093015

**Published:** 2018-09-09

**Authors:** Sumit Gupta, Han-Joo Lee, Kenneth J. Loh, Michael D. Todd, Joseph Reed, A. Drew Barnett

**Affiliations:** 1 Department of Structural Engineering, University of California-San Diego, La Jolla, CA 92093-0085, USA; sggupta@eng.ucsd.edu (S.G.); mdtodd@ucsd.edu (M.D.T.); 2 Material Science and Engineering Program, University of California-San Diego, La Jolla, CA 92093-0085, USA; hal257@eng.ucsd.edu; 3 Elintrix, Escondido, CA 92029, USA; jreed@elintrix.com (J.R.); dbarnett@elintrix.com (A.D.B.)

**Keywords:** carbon nanotube, electrical capacitance tomography, nanocomposite, noncontact, osseointegrated prosthesis, patterning, permittivity, strain sensing, thin film

## Abstract

The objective of this study was to develop a noncontact, noninvasive, imaging system for monitoring the strain and deformation states of osseointegrated prostheses. The proposed sensing methodology comprised of two parts. First, a passive thin film was designed such that its electrical permittivity increases in tandem with applied tensile loading and decreases while unloading. It was found that patterning the thin films could enhance their dielectric property’s sensitivity to strain. The film can be deposited onto prosthesis surfaces as an external coating prior to implant. Second, an electrical capacitance tomography (ECT) measurement technique and reconstruction algorithm were implemented to capture strain-induced changes in the dielectric property of nanocomposite-coated prosthesis phantoms when subjected to different loading scenarios. The preliminary results showed that ECT, when coupled with strain-sensitive nanocomposites, could quantify the strain-induced changes in the dielectric property of thin film-coated prosthesis phantoms. The results suggested that ECT coupled with embedded thin films could serve as a new noncontact strain sensing method for scenarios when tethered strain sensors cannot be used or instrumented, especially in the case of osseointegrated prostheses.

## 1. Introduction

Limb amputation is an unfortunate but necessary consequence of those who are involved in severe trauma, vascular diseases, or certain cancers [1]. In the United States, more than 2 million people suffer from limb loss, with ~185,000 amputations performed every year [2]. Nevertheless, a vast amount of research is dedicated to devising technologies that can help improve the post-amputation quality of life of amputees. Recent advancements in prosthetic technologies have made significant progress in helping amputees to regain their lost functionality due to limb amputation. For example, lower extremity amputation is generally treated by attaching a prosthesis to the residual limb by means of a customized socket. This type of prosthesis often causes discomfort to the patients and, in extreme cases, leads to skin irritation and pressure ulcers. In addition, quality of life can be affected as a result of limited force transfer and motor control, as studied by Pitkin et al. [3].

Since the 1990s, osseointegrated prostheses (OIP) have been investigated as a potential alternative to conventional socket-type prostheses. OIPs offer unrestricted ranges of motion, improved sensory feedback through osseo-perception, and, last but not least, better sitting comfort with reduced soft-tissue problems [4]. Osseointegration is defined as a direct functional and structural connection between the bone and the surface of the load-carrying implant [5]. It is achieved by a two-stage surgical process, where a metal implant is inserted into the bone in the residual limb. Then, a second surgery is performed after six to eight weeks of the first surgery; a small skin incision is made, and a connection between the abutment and the osseointegrated prosthesis is achieved. After a week of the second surgery, the patients need to undergo rehabilitation, where they are trained to progressively load the prosthesis and to promote osseointegration between the implant and bone. The rehabilitation period can range from 6 to 12 months [6].

The rehabilitation program comprises a combination of dynamic and static load bearing exercises (LBE), where it is hypothesized that the timely application of a suitable amount of strain on the implant would help to simulate bone formation around the prosthesis and prepare the implant to withstand mechanical loading likely to occur during daily activities. In general, patients load the prosthesis twice a day for ~30 min [7]. It was reported by several researchers that different mechanical loads, rates of applied strain, strain distributions, and number of strain cycles, to name a few, are important to influence the rate of bone growth around the tissue-prosthesis interface [8,9]. At the same time, while a minimum effective strain is required to activate the bone adaptation process, overloading may place the bone-implant interface at risk [10]. Thus, it is evident that the ability to accurately measure implant strains and stresses would help doctors and clinicians better understand the effects of different strain-states on bone-remodeling, ascertain the degree of osseointegration, and design a more efficient rehabilitation process for the patient. The ability to monitor strains also means that clinicians and patients can determine whether suitable amounts of strains are applied to the implant during LBEs [8]. In addition to monitoring uniaxial strains on the OIP, bending or flexural strains are also of interest. Excessive bending during extreme events (e.g., a fall) would result in prosthesis loosening and failure, especially during rehabilitation [11]. It is found that half of the patient population with OIPs experience a fall at least once a year [12].

Currently, the mechanical loading applied during static LBE is generally monitored using a bathroom scale, which provides immediate visual feedback, but its application is limited as it can only measure the vertical component of applied load [8]. Assessment of the applied force on the implant during LBE can also be performed with the aid of advanced inverse dynamic method used in a motion analysis system laboratory [13]. A force plate instrumented with external force measuring transducers is generally employed to directly measure the forces experienced by the implant during rehabilitation [14]. One of the main drawbacks of this method is that only the first few steps of walking can be accurately measured. This method can also result in an altered gait, thereby limiting the accuracy of the calculated load. On the other hand, it is difficult to simulate other daily activities in these laboratory environments [15].

To overcome these aforementioned limitations, attempts have been made to directly mount a load sensor onto the prosthesis. Frossard et al. [16] presented a kinetic portable system that relies on a commercial transducer to measure the actual load applied to the distal end of the implant. They successfully recorded the triaxial forces and moments applied to the implant. However, these transducers are point-sensors that require multiple transducers for estimating the load distribution over the entire prosthesis. Each transducer would also require lead wires connected to a separate sensing channel, and this requirement can make it difficult for practical implementations because of the number of tethered connections needed. Thus, a wireless modem was investigated for directly measuring and transferring data to a centralized data repository [17]. Such a wireless measuring system adds fidelity to record reliable and accurate measurements of load during daily activities. The iPecsTM load cell is a wireless unit that was used to measure three-dimensional (3D) forces and moments in OIPs [18]. However, in vivo measurement of strains in OIPs remains extremely challenging, because most of the transducers need to be mounted on the external surface of the implant, where it is almost impossible to mount strain gages onto the prosthesis while it is embedded in the patient’s body. Besides, as a rigid connection between the implant and transducers is one of the primary requirements for accurately measuring force or implant strains, this may cause patient discomfort.

Therefore, the main objective of this study is to develop a noncontact, noninvasive sensing technique that can be potentially used for monitoring in situ strains in OIPs. Achieving this goal would help improve the basic understanding of bone remodeling and ultimately provide better clinical guidance during rehabilitation. Furthermore, the potential of extending this technology to become wearable suggests that real-time OIP strain monitoring could be realized in the future. The method proposed involves two main parts. First, a new type of multi-walled carbon nanotube (MWCNT)-based thin films, whose electrical permittivity is sensitive to strain, were designed and fabricated. Uniform and patterned thin films were tested to characterize their sensing properties. Second, to realize noncontact strain sensing, an electrical capacitance tomography (ECT) measurement technique and algorithm were implemented. Using ECT, permittivity distribution could be reconstructed from boundary capacitance measurements. Then, to validate noncontact strain sensing, thin films were deposited onto prosthesis phantoms and subjected to different loading patterns, while ECT was used to interrogate the system at different strain states. The preliminary results showed that the nanocomposite thin film used in conjunction with ECT could quantify the strain- and deformation-states of OIP phantoms in a noncontact fashion. The research significance is the demonstration of noncontact strain sensing using ECT interrogating a passive nanocomposite element deposited where strain measurements are desired.

## 2. ECT Background

**Figure 1:**
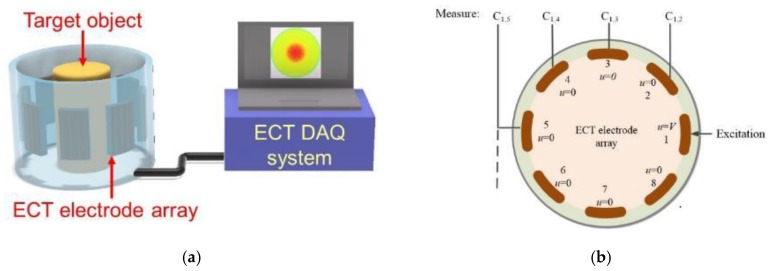
(**a**) The electrical capacitance tomography hardware system is illustrated. (**b**) The cross-section of the ECT electrode array is shown with its electrode numbering scheme. A typical ECT excitation-measurement sequence is also depicted.

ECT is a soft-field imaging technique that can estimate the permittivity distribution of a predefined sensing region [19]. A series of equidistantly spaced electrodes are arranged along the perimeter of a circular sensing region. An electrode is excited with an alternating current (AC) signal, while the capacitance between the excitation and other electrodes are measured. The electrode used for excitation is switched, and capacitance measurements at all other electrodes are acquired. This process is repeated until every electrode is excited. An ECT data acquisition (DAQ) test setup with a typical excitation and measurement pattern is illustrated in Figure 1. Electrical permittivity distribution inside the circular sensing region can then be reconstructed from this measured set of capacitances by solving the ECT inverse problem. 

Since the development of ECT in the 1980s [20], it has mainly been used for flow monitoring in applications such as gas separation [21]; pneumatic conveyors [22]; and, more recently, fluidized bed dryers [23]. ECT has also been used for monitoring other industrial processes (e.g., visualization of flame during combustion [24], nylon-polymerization reaction monitoring [25], and curing monitoring of epoxy resins [26], among others). In addition, ECT was recently used as a nondestructive evaluation (NDE) tool to assess the subsurface condition of structures [27,28]. Apart from these structural applications, ECT is also used in biomedical imaging. A dual modality imaging technique, using ECT and electrical impedance tomography (EIT), was proposed by Ren et al. [29] to provide real-time 3D images to navigate surgery tools in the femoral bone during total hip replacement. Taruno et al. [30] suggested a real-time, volumetric, capacitance-based imaging technique for monitoring brain activities related to human motoric and lung functions. In addition to these applications, previous work by Gupta and Loh [31] showed the ability of ECT, when coupled with an embedded pH-sensitive thin film, to monitor infection at OIP-tissue interfaces. It was also shown that ECT could be used as a noncontact, radiation-free, imaging tool for monitoring prosthesis loosening and bone fracture at bone–OIP interfaces [32]. The mathematical formulation of ECT is briefly discussed in the following section, and more details can be found in Gupta and Loh [31].

### 2.1. ECT Forward Problem

The ECT forward problem computes the boundary capacitance response of a sensing domain (Ω) given *a priori* knowledge of the electrical permittivity distribution in Ω. The ECT forward problem can be defined by 2D Laplace’s equation:

(1) ∇⋅(ε∇u)=0

where *ε* and *u* are the electrical permittivity and electric potential distribution inside Ω, respectively. This second order-partial differential equation needs to be solved with proper boundary conditions to determine *u* everywhere in Ω. In practice, finite element modeling (FEM) is often employed to solve Equation (1). Once *u* is calculated, Equation (2) can be used to estimate the mutual capacitance (*C_i,j_*) between the excitation (*i*) and sensing electrodes (*j*):

(2) $$C_{i,j}=\frac{1}{V}\int_{e_{j}}\varepsilon \frac{\partial u_{i}}{\partial n}dl\;i=1:L;\;j=i+1:L$$

where *n* is the unit inward normal to the *j*^th^ measurement electrode, *e_j_* is the length of that same electrode, *V* is the magnitude of applied voltage, *L* is the total number of electrodes in the electrode array, and *u_i_* is the solution of the forward problem when the *i*^th^ electrode is used for excitation.

### 2.2. The Corresponding Inverse Problem

While the ECT forward problem is solved to estimate the boundary capacitance responses (*C_c_*) for an assumed permittivity distribution of Ω, an inverse problem is formulated and implemented to reconstruct the permittivity distribution from the measured set of capacitances (*C_m_*). Because ECT is an ill-posed and nonlinear inverse problem, regularization must be incorporated into the problem formulation. In this study, a Gauss–Newton iterative algorithm with total variation (TV) regularization [33] was implemented to minimize the error norm between computed and measured sets of capacitances to estimate the permittivity distribution of Ω. TV regularization in its continuum form is shown in Equation (3).

(3) $$TV(\varepsilon )=\int_{\Omega }\left|\nabla \varepsilon \right|\;dxdy$$

In general, the inverse algorithm starts with an initial assumption of permittivity distribution, and *C_c_* is calculated by solving the forward problem. The measured set of capacitances is used to evaluate the difference (*e*) between *C_m_* and *C_c_*:

(4) $$e=C_{m}-C_{c}$$

A new permittivity distribution is then estimated by solving the inverse problem, and this process is repeated until the ratio (*ϵ*) of the norm of *e* and the norm of *C_m_* remains greater than a preset error threshold (0.05%). A detailed formulation of the ECT forward and inverse problems can be found in Gupta and Loh [31] and Soleimani and Lionheart [19].

## 3. ECT Validation and Resolution Characterization

**Figure 2:**
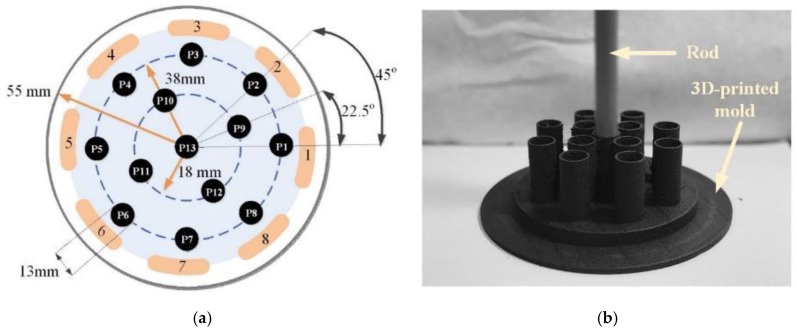
(**a**) To characterize ECT permittivity imaging accuracy, a 13 mm-diameter polyvinyl chloride (PVC) rod was placed at 13 different locations in the ECT electrode array. Their positions were marked as P1, P2, P3, …, and P13. (**b**) A 3D-printed mold was prepared and employed to accurately maintain the locations of the rod, and the mold was fitted below the ECT electrode array.

**Figure 3:**
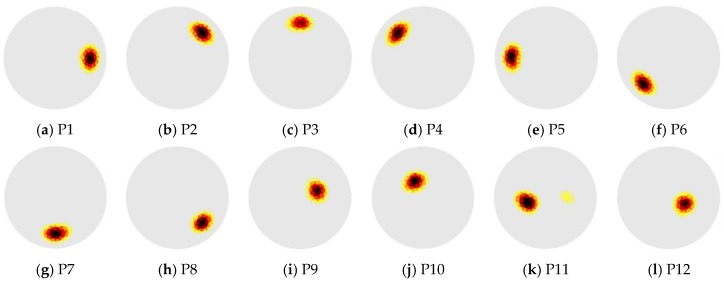

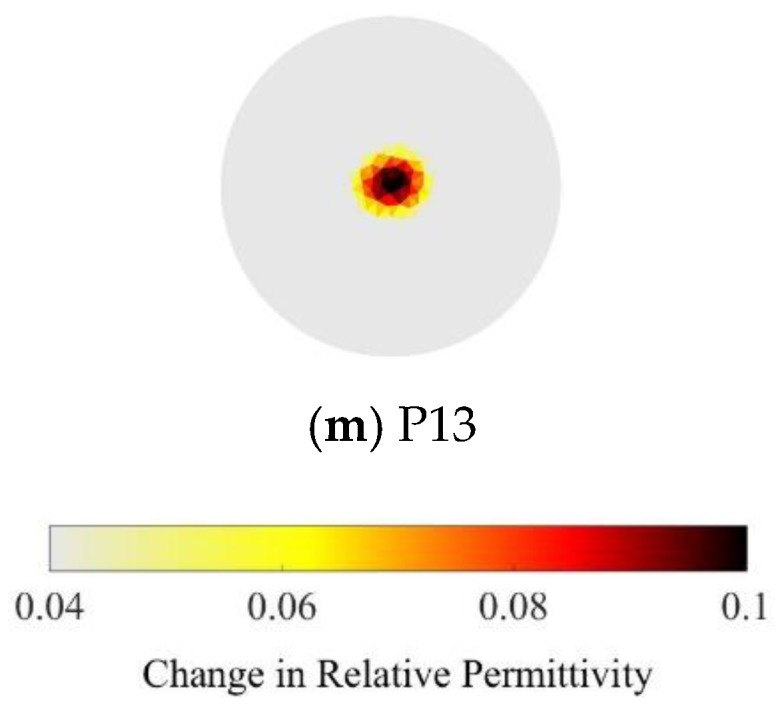
The ECT images were determined when the PVC rod was placed at (**a**) P1, (**b**) P2, …, to (**m**) P13.

It has been observed in previous studies that reconstructed ECT images show spatial non-uniformity in terms of image amplitudes, positions, and resolutions. These uncertainties could make ECT images difficult to interpret and prone to error. Hence, the first part of this work focused on performing a qualitative evaluation of ECT reconstruction. An FEM model of the sensing region was created and discretized into 4000 linear triangular elements. For the experiment, a 13 mm-diameter polyvinyl chloride (PVC) rod was placed at 13 different locations in the sensing area (i.e., from P1 to P13) as shown in Figure 2a. In order to accurately maintain the positions of the rod, a customized holder, shown in Figure 2b, was fabricated using a Type A Series 1 Pro 3D printer. This holder was fitted below the ECT electrode array and served as a receptacle for the PVC rod, where the rod could be fitted in any of the 13 positions. Prior to putting the rod in the ECT electrode array, a set of boundary capacitances was obtained with the ECT electrode array empty and filled with air. Here, the excitation was a 15 V, peak-to-peak, 1.25 MHz AC, square-wave signal. The inverse problem was then solved, and the corresponding reconstructed permittivity distribution was used as the baseline. Next, the rod was placed at the designated locations, and ECT measurements were obtained for permittivity reconstruction. The final permittivity distributions were obtained by subtracting out the previous baseline, and the results are shown in Figure 3. It can be observed from Figure 3 that the ECT inverse algorithm was able to detect the location and size of the PVC rod for all 13 cases from P1 to P13.

### 3.1. Resolution Study

After successfully detecting the locations of the PVC rod in the ECT electrode array, different image parameters were computed to quantitatively assess the quality of reconstructed images. A series of different image parameters were proposed by Adler et al. [34] to study the image quality of EIT images. In this work, three of these evaluation criteria were adopted and implemented for ECT image quality analysis. First, the one-fourth amplitude set or [*ε_q_*]*_i_*, which contains all the image pixels ([*ε*]*_i_*) greater than one-fourth of the maximum amplitude of all the image pixels, was determined. Mathematically, [*ε_q_*]*_i_* can be expressed as follows:

(5) [εq]i={1if [ε]i≥14max[ε]0otherwise

where [*ε*] is the reconstructed permittivity distribution. Position error (*PE*) was estimated by calculating the distance between the center of gravity (CG) of the target object (i.e., the PVC rod) and the CG of [*ε_q_*]*_i_*. *PE* measures the extent to which reconstructed images accurately represent the position of the target object and is computed as follows:

(6) $$PE=r_{t}-r_{q}$$

where *r_t_* and *r_q_* are the distances of the CG of the target object and [*ε_q_*]*_i_*, respectively, where both are with respect to the center of the ECT electrode array. Second, to estimate the resolution (*RES*) of the reconstructed images, the square root of the ratio of the area (*A_q_*) corresponding to [*ε_q_*]*_i_* and the total sensing area (*A*_0_) was evaluated [35]:

(7) RES=AqA0

Last, shape deformation (*SD*) factor is defined as the fraction of the reconstructed one-fourth amplitude set that does not fit into a circle (*C*) of equal area:

(8) SD=∑i∉C[εq]i∑i[εq]i

### 3.2. Resolution Study Results

**Figure 4:**
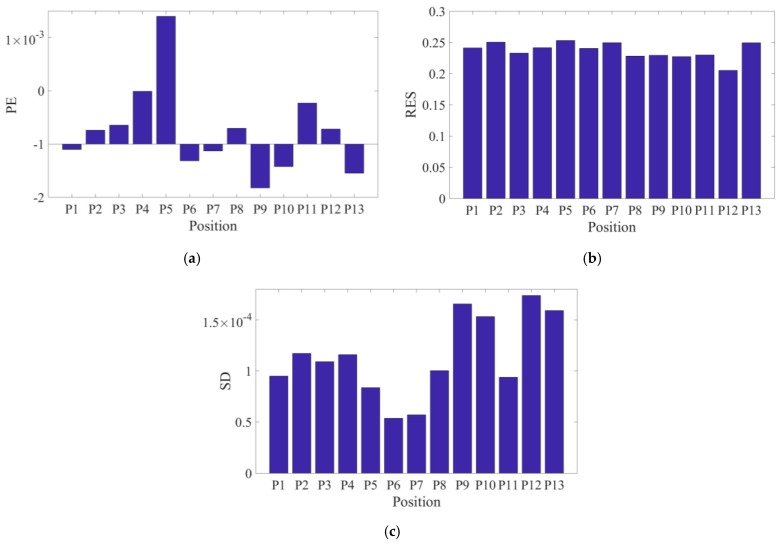
The results of (**a**) position error, (**b**) resolution, and (**c**) shape deformation of the reconstructed images are plotted with respect to each of the P1 to P13 test cases.

These three performance parameters (i.e., *RES*, *PE*, and *SD*) were computed for all 13 rod-position cases from P1 to P13 (Figure 2a) to evaluate the performance of the ECT algorithm. Figure 4 shows each of the evaluation criteria results calculated for each rod position, while Table 1 summarizes the statistical properties of each criterion considering all positions. In general, smaller standard deviations indicate better accuracy of reconstructed images in terms of the rod’s location, shape, and size. Even though the magnitudes of *PE* are very small over the entire ECT electrode array (Figure 4a), their bipolar distribution suggests that reconstructed images are sometimes pushed towards or away from the center of the sensing domain. The slightly larger values of *RES* indicate that the ECT algorithm tends to overestimate the size of the target object (Figure 4b). The smoothing effect of the regularization scheme used in this study may contribute to this overestimation. Despite this, the incredibly small values of *SD* (i.e., on the order of 10^−4^) and its small standard deviations indicate the low risk of incorrectly interpreting the reconstructed permittivity maps, thereby successfully validating its position-detection robustness. In summary, these results successfully validated the ECT system, where the locations, shapes, and sizes of permittivity changes was visualized fairly accurately.

## 4. Noncontact Strain Monitoring

**Figure 5:**
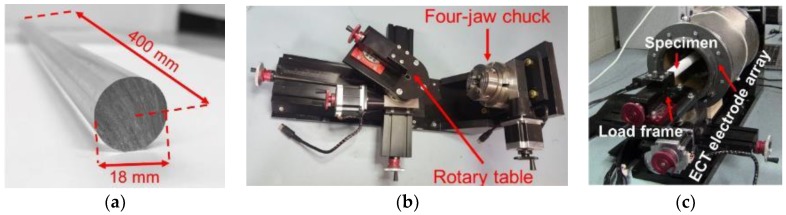
(**a**) In this study, 18 mm-diameter, 400 mm-long plastic rods were used as the test structure and were subjected to different types of loading. (**b**) A customized prototype load frame was designed and built by Elintrix. (**c**) The specimen was fitted into the load frame with the ECT electrode array for testing.

After quantifying the accuracy of the ECT inverse algorithm, a set of experiments was performed to verify if deformation in the prosthesis phantom could be monitored in a noncontact fashion via ECT. A 20 mm-diameter plastic rod (Figure 5a) was used as the prosthesis phantom and subjected to uniaxial and bending loads, while its change in electrical permittivity due to strain/deformation was monitored with ECT. Although a plastic rod was used, it could be replaced with a metallic specimen. A previous study by Gupta and Loh [36] demonstrated that ECT could also detect strain-induced permittivity changes of a nanocomposite-coated metallic prosthesis phantom.

**Figure 6:**
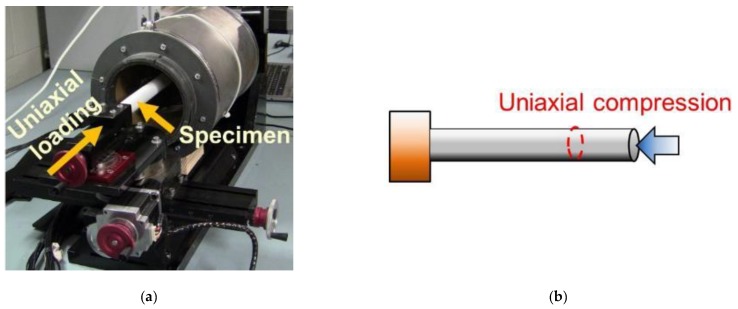
(**a**) The picture of the load frame and (**b**) the schematic shows that uniaxial compressive load was applied to the test specimen, and ECT measurments were recorded at different strain states.

**Figure 7:**
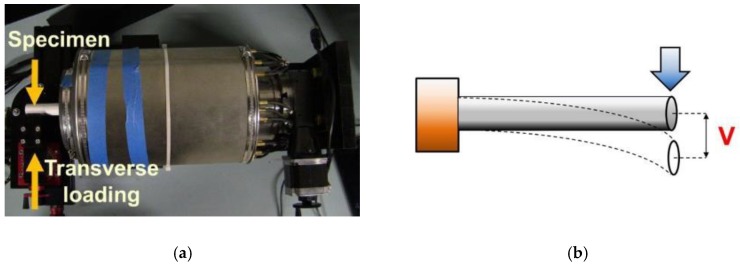
(**a**) The picture of the load frame and (**b**) the schematic shows that transverse loads were applied to the specimen to induce bending.

In this study, a customized load frame (designed and built by Elintrix), shown in Figure 5b, was configured to apply different types of loads (i.e., uniaxial compression and bending) to an OIP surrogate. First, the surrogate was fitted in the load frame via a four-jaw-chuck so that one end was completely fixed. Second, the ECT electrode array was slipped over the 400 mm-long rod such that the rod was located at the center of the ECT electrode array cross-section, as shown in Figure 5c. Next, two sets of experiments were performed. The first set of tests subjected the phantom to uniaxial one-cycle compressive loading and then unloading, as shown in Figure 6. The second set of tests subjected the specimen to transverse displacements (*V*) to cause bending, as shown in Figure 7. A major benefit of the Elintrix load frame was the fact that one of its four-jaw adjustable grips was fitted onto a rotary table, thereby allowing the load frame to apply mixed-mode loading to the specimen (i.e., bending, compression, or a combination). It should be mentioned that, prior to the application of any load to the specimen, a baseline set of capacitances was obtained, and the corresponding permittivity distribution (i.e., baseline) was reconstructed.

The change in permittivity distributions of different strain states with respect to the baseline (i.e., the undeformed state) was determined. It was found that no significant change in electrical permittivity of the rod was observed in the entire sensing domain. The specimen was also subjected to cantilevered bending loads, and ECT measurements were recorded in a similar way. For all these tests, ECT was unable to detect any significant changes in electrical permittivity of the specimen due to applied strains. On the basis of these findings, the conclusion is that strain-induced changes in electrical permittivity of the pristine specimen (i.e., OIP phantom itself) were too small to be captured by ECT, and, hence, alternatives are needed.

## 5. Strain-Sensitive Nanocomposite Thin Films

Because the change in electrical permittivity of the prosthesis surrogate was not significant enough to be captured by ECT, an objective of this study was to design a nanocomposite thin film whose dielectric property is sensitive to strain and deformation. Among the variety of nanomaterials that exist today, carbon nanotubes (CNT) have drawn significant attention because of their superior electromechanical properties. In fact, many researchers successfully demonstrated that CNTs could be effectively integrated into polymer matrices to realize high-performance thin film strain sensors [37]. In addition, CNTs were also used for various biomedical applications. For example, by stimulating the production of the extracellular matrix during formation of bone tissues, CNTs could escalate the growth of osteoblastic cells in the human body [38]. A separate study performed by Zhang et al. [39] showed that chemically modified MWCNTs did not have any detrimental effects on human osteoblasts MG-63 cells. More recently, it was demonstrated that MWCNTs could stimulate the growth of inducible cells in soft tissues to form inductive bone by concentrating more proteins [40]. However, the hydrophobic nature of CNTs limits their biomedical applications. Therefore, different polymers have been used as a dispersing medium to prevent agglomeration of CNTs in aqueous solutions. For instance, poly(sodium 4-styrenesulfonate) (PSS) was identified as a biocompatible polymer that can also promote cellular adhesion by means of electrostatic interactions [41].

The nanocomposite used in this study was an MWCNT and PSS-based thin film that can be deposited onto the surface of OIPs by spray coating [42], and they served as a passive sensor whose strain-induced permittivity changes could be quantified (in a noncontact manner) by ECT. Furthermore, this study also investigated the effects of patterning on strain sensitivity. When subjected to strain and deformation, these stacks of patterned films change their dimensions, thus leading to intensified local changes in electrical permittivity distribution. As the change in dielectric property gets magnified as a result of the incorporation of this strain-sensitive thin film, ECT can be employed to detect the permittivity change during deformation in a noncontact fashion.

### 5.1. Thin Film Fabrication

The formulation of the MWCNT-latex thin film used in this study was based on studies reported by Loyola et al. [43] and Mortensen et al. [42]. In short, MWCNTs were dispersed by mixing them in a 2 wt.% PSS aqueous solution followed by subjecting the mixture to high-energy probe sonication. A sprayable ink was then obtained by adding an appropriate amount of Kynar Aquatec latex solution to the dispersed MWCNT–PSS solution, which was then directly sprayed using an airbrush to deposit the films. Mortensen et al. [42] used scanning electron microscopic (SEM) images to characterize the morphology of 30 MWCNT–PSS/latex thin films deposited onto glass microscope slides. It was found that MWCNTs were uniformly and randomly distributed in the PSS–latex matrix. The average film thickness was found to be ~10 µm. It was suggested that thermal annealing could be employed to further improve their electromechanical properties.

For all types of specimens tested in this study, a thin layer of paint primer was first deposited onto the surface of the substrate to serve as an electrically insulating layer and to ensure that the film adheres to the substrate. While paint primer is obviously not biocompatible, future studies will investigate other biocompatible thin film precursor alternatives that could be implemented on OIPs. Nevertheless, once the primer fully dried, the MWCNT–latex film was spray-coated. Two different types of films were fabricated, which are herein referred to as Thin film-A and Thin film-B. Both films were MWCNT–latex films but were deposited differently to create specific patterns. First, Thin film-A was created by spray-coating a uniform MWCNT–latex film. After the film completely dried, another layer of insulating primer was deposited on top of it.

**Figure 8:**
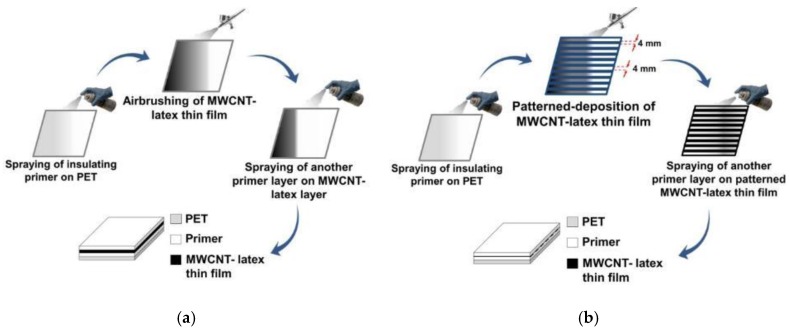
The fabrication procedure for (**a**) Thin film-A and (**b**) -B are illustrated. MWCNT—multi-walled carbon nanotube; PET—polyethylene terephthalate.

Second, Thin film-B was a patterned thin film. The hypothesis was that patterning could enhance the electrical permittivity’s sensitivity to applied strains. Using the primer-coated substrate, 4-mm wide strips of masking tape were applied to form a multi-striped pattern, with each mask separated by a 4 mm gap from its adjacent ones. Then, a layer of MWCNT–latex thin film was deposited, followed by removal of the masks to leave behind patterned strips of nanocomposites. The final step entailed spraying another layer of primer over the entire patterned film. Effectively, the alternating strips of conductive MWCNT-latex films and insulating layers served as a compound parallel-plate capacitor, which changed its perceived capacitance and permittivity due to strain, thus enhancing its strain sensing performance. The fabrication procedures of Thin films-A and -B are schematically shown in Figure 8a,b, respectively. It should be mentioned that spray-coating can be performed manually or using a robotic spray fabrication system.

### 5.2. Strain Sensing Characterization

**Figure 9:**
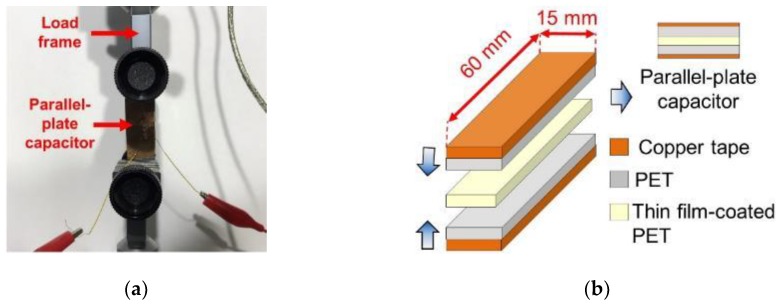
(**a**) A nanocomposite-coated PET strip was mounted in the load frame for characterizing its strain sensitivity. (**b**) Copper tape electrodes were used to interrogate the capacitance response of the film-coated PET.

To characterize how the nanocomposites’ permittivity responded to applied strains, Thin films-A and -B were deposited onto primer-coated polyethylene terephthalate (PET) sheets, and a parallel-plate capacitor test setup was employed. First, the film-coated PET was cut into a rectangular strip and mounted in a Test Resources 150R load frame. Second, using two separate (and pristine) PET strips of identical size, conductive copper tape was affixed onto one side of each of those PET strips. These copper tape–PET strips were then mounted adjacent to the film-coated PET specimen and clamped in the load frame (Figure 9a). Such an arrangement formed a parallel-plate capacitor with the film-coated PET (and additional layers of PET) as the dielectric medium between the two copper tape electrodes. This setup also ensured that the electrodes are not strained when the film-coated PET was loaded. The test setup and its assembly processes are illustrated in Figure 9b.

Using this test setup, the load frame was commanded to apply uniaxial tension to the specimen at a constant strain rate (588 *με*/min) from 0 to 4902 *με*, pausing every 980 *με* for capacitance measurements using a Keysight E4980A LCR meter. This was followed by unloading the specimen to 0 *με* and pausing accordingly for capacitance measurements. It should be mentioned that the LCR meter applied a 1 V, peak-to-peak, 1.25 MHz sinusoidal excitation signal; capacitance was also measured in parallel-circuit mode (*Cp*), because the impedance of all the specimens was greater than 10 kΩ. Five specimens of each type were prepared and tested to study the dielectric behavior of Thin films-A and -B when subjected to tensile loading and unloading. In a separate study done by Wang et al. [44], the piezoresistive behavior of the nanocomposite thin film was characterized at a higher strain rate (~5000 *µε*/min). It was found that the electromechanical response of the thin film was still linear; hence, this film could serve as a robust strain sensor even at higher applied strain rates.

**Figure 10:**
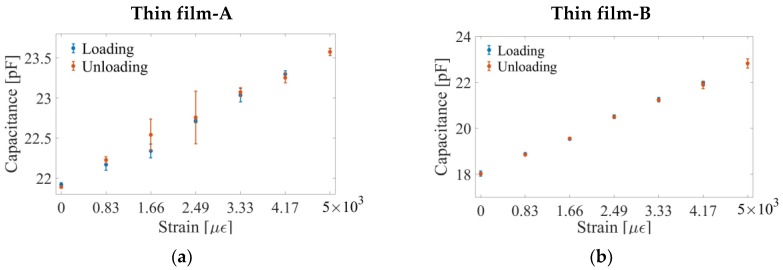
The capacitance of the film-contained parallel-plate capacitor during loading and unloading are shown for (**a**) Thin films-A and (**b**) -B. Standard deviations of measured capacitances are also shown (based on five specimens tested).

Figure 10a,b show the average capacitances of the Thin film-A and Thin film-B parallel-plate capacitors, respectively, during loading and unloading. Although the results shown here were obtained from only one loading cycle, it can be observed that the capacitance corresponding to the same strain-state during loading and unloading is nearly identical, which demonstrated their repeatability and suitability for strain sensing. However, it is important to study the long-term electromechanical behavior of the nanocomposite thin film to establish this thin film as a reliable strain sensor for OIP monitoring. In a previous study done by Wang and Loh [45], 5-, 10-, and 15-tensile cyclic strain patterns were applied, and the resistance of the film was simultaneously measured. Although the thin film exhibited cyclic changes in its electrical property in tandem with the applied cyclic strain pattern, the nominal resistance of the film drifted, and it took a very long time to stabilize (>30 min). Thermal annealing could be applied to avoid such drifts in nominal resistance of the thin film.

**Figure 11:**
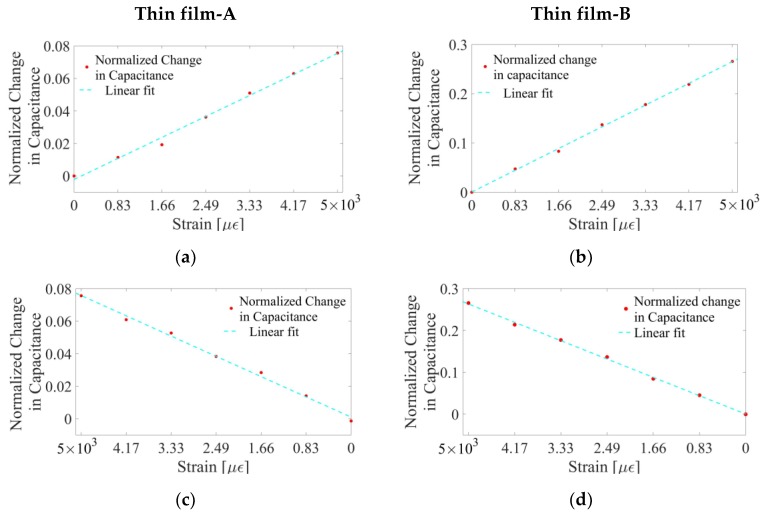
(**a**) Thin films-A and (**b**) -B were stretched, and the change in capacitance with respect to their undeformed states are shown, respectively. The change in capacitance during the unloading cycle for (**c**) Thin films-A and (**d**) -B are shown, respectively. Strain sensitivity of the Thin films-A and -B was found to be ~1.58 × 10^−5^ and ~5.37 × 10^−5^, which was based on the slope of the linear best-fit line.

To better compare the capacitance results of different specimens, normalized capacitance (*C_N_*) was computed as follows:

(9) $$C_{N}=\frac{\Delta C_{i}}{C_{0}}$$

where *C*_0_ is the film’s capacitance prior to applying any strains, and Δ*C_i_* is the change in capacitance of the specimen (at any given strain state, *i*) with respect to *C*_0_. Representative results for Thin films-A and -B during loading and unloading are plotted in Figure 11. From the results shown in Figure 11, it is evident that both types of films exhibited strain-sensitive-dielectric behavior, where their capacitance increased in tandem with increasingly applied tensile strains and decreased when load was gradually removed.

It was also observed that the strain sensitivity of Thin film-B was ~4 times higher than that of Thin film-A, and this was true for both loading and unloading. Here, the strain sensitivity of these experimental results can be assumed to be equal to the slope of the linear least-squares best-fit line. Thus, it can be concluded that patterning the conductive MWCNT–latex thin film could enhance strain sensitivity. The patterns can be optimized for maximizing strain sensitivity, but this will be done in the future.

## 6. Noncontact Strain Monitoring using Patterned Films

### 6.1. Uniaxial Loading

**Figure 12:**
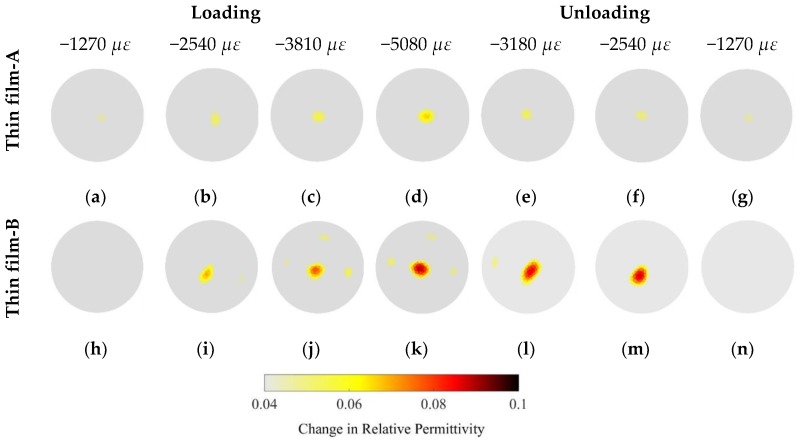
(**a**–**d**) show the changes in electrical permittivity of the Thin film-A-coated test specimen subjected to uniaxial compressive strain, while (**e**–**g**) describe the change in electrical permittivity during unloading. The Thin film-B-coated specimen was also subjected to the same loading conditions, and its changes in permittivity are shown during loading (i.e., (**h**–**k**)) and unloading (i.e., (**l**–**n**)).

It was mentioned earlier that ECT was unable to detect any significant changes in electrical permittivity caused by the deformation of the pristine test specimen (i.e., OIP surrogate alone). Therefore, strain-sensitive Thin films-A and -B were deposited onto the surfaces of the test specimens and were subjected to the same uniaxial compressive loading conditions as before. ECT measurements were obtained at the same strain levels, and the corresponding changes in their electrical permittivity distributions with respect to their undeformed states are shown in Figure 12. It can be observed from these results that both of the thin film-coated specimens exhibited electrical permittivity changes as they were loaded. Permittivity changes occurred where the rod was physically located in the sensing domain (i.e., in the center). Some permittivity changes were also observed near the boundary regions, but this was likely due to measurement and environmental noise that corrupted capacitance measurements and influenced ECT reconstruction results. Second, the magnitudes of permittivity changes as a result of applied strains as determined by ECT (Figure 12) were comparable to the aforementioned parallel-plate capacitor tests (Figure 11). Because the strain sensitivity of Thin film-A was lower than that of Thin film-B, it can be seen in Figure 12 that the signal-to-noise ratio is higher (i.e., the magnitude of the change in electrical permittivity is larger) for the patterned specimen (Thin film-B). 

### 6.2. Cantilever Bending

**Figure 13:**
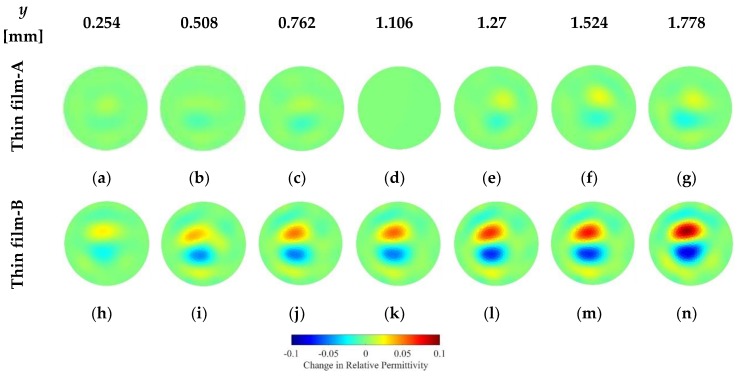
Thin films-A and -B coated onto osseointegrated prostheses phantoms were subjected to transverse loading. The corresponding changes in electrical permittivity with respect to the undeformed states are shown. (**a**–**g**) describes the permittivity changes for the specimen coated with Thin film-A, while (**h**–**n**) illustrate the electrical permittivity changes of the Thin film-B-coated specimen.

It is known that bending of a cantilever beam induces tension in one side with respect to the neutral axis and compression in the other. In this study, the custom Elintrix load frame was adjusted to apply transverse displacements (*y*) or loads to the free-end of the film-coated OIP phantoms, as was discussed earlier. Loading was paused at different displacement intervals to allow the ECT system to interrogate the sensing region and the specimen. Figure 13 shows a representative set of results. Although no significant changes in electrical permittivity were observed near the central region when a Thin film-A-coated specimen was subjected to bending (first row of Figure 13), a bipolar change in electrical permittivity was observed in the Thin film-B-coated specimen (bottom row of Figure 13). The reason that only the Thin film-B-coated specimens produced a response was because of its higher strain sensitivity and larger signal-to-noise-ratio. The bipolar response was also expected (i.e., given that the rod was simultaneously experiencing tension and compression), and these results validated the fact that the film could sense both tensile and compressive strains when coupled with ECT. However, the size of the specimen was exaggerated by ECT, which could be because of the particular regularization scheme employed. This is also expected because previous tests revealed larger *RES* errors in the reconstructed images. 

It should be mentioned that the ECT electrodes have a finite length (~35 mm). This means ECT would output an average of any strain changes occurring over the interrogated region of the cantilevered OIP phantom. This effect can be mitigated by redesigning the electrode array to feature shorter electrodes. Another possibility is to move the electrode array along the length of the specimen to determine average strain-induced permittivity changes over a given segment. Such an approach can lead to the generation of 3D ECT-reconstructed permittivity distributions, which is the subject of future investigations. Overall, these results successfully demonstrated noncontact strain monitoring.

**Figure 14:**
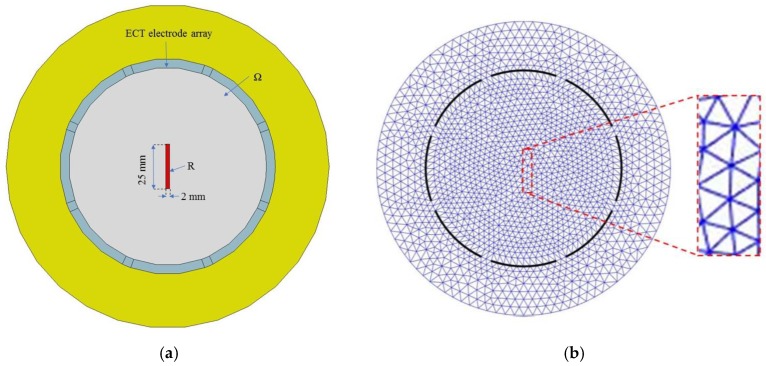
(**a**) A 25 mm-long, 2 mm-wide rectangular region was considered at the center of the sensing region and coincided with a portion of the OIP phantom. (**b**) The rectangular strip consisted of 18 triangular elements.

The data from Figure 13 were also further processed to reveal the change in electrical permittivity at different depths of the OIP surrogate’s cross-section. To do this, a 25 mm-long, 2 mm-wide rectangular section (R) of the OIP specimen was considered (Figure 14a), and its center coincides with the center of the OIP rod in the previous test. From the FEM, it was found that *R* bounded 18 triangular elements spanning two columns (Figure 14b). The permittivity values of these elements in *R* were extracted, corresponding to the different stain states tested. Here, only data for the Thin film-B-coated specimen was processed because of its greater sensitivity to strain.

**Figure 15:**
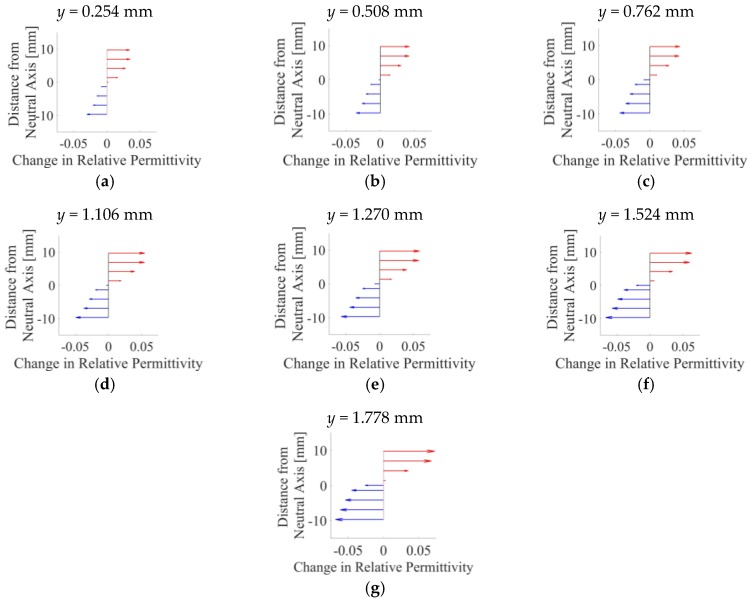
The change in electrical permittivity in R at different distances from the neutral axis position (i.e., the center of the OIP surrogate) corresponding to different *y* are plotted in (**a**–**g**).

Figure 15 plots how the electrical permittivity of the Thin film-B-coated specimen varied as a function of depth. First, the results show that strain varied almost linearly, from tension (positive strain) in the upper portion to compression (negative strain) in the lower section of the OIP phantom. Second, the permittivity change is close to 0 near the neutral axis (i.e., the center of the rod), which is expected, while gradually increasing and moving towards the extreme values near the top or bottom. Third, the maximum absolute change in electrical permittivity increased as *y* was gradually increased from 0.254 to 1.778 mm, which is shown by the length of the top and bottom arrows in Figure 15. The linear change in electrical permittivity over the cross-section of the specimen shown in Figure 15 resembles the same strain distribution pattern as one would expect for a cantilevered beam subjected to bending. Overall, these results also show that the bending strain distribution over the cross-section of a nanocomposite-coated prosthesis surrogate could be captured with the aid of ECT.

### 6.3. Significance of Results

The significance of this study is the successful validation of noncontact strain sensing using ECT coupled with a passive nanocomposite element deposited where strain measurements are desired. The incorporation of a passive thin film sensing element enabled noncontact sensing of a specific stimulus, in addition to mapping the cross-sectional electrical permittivity of the OIP surrogate. Previous work leveraged this principle, but focused on using ECT for quantifying pH changes (as a precursor of infection [31]), as well as for assessing prosthesis loosening and bone fracture (but, in this case, without using an embedded thin film [32]). In contrast, this study entailed the design, fabrication, and characterization of a new nanocomposite whose electrical permittivity responded to applied strains. The thin film sensing element’s design and formulation was new when compared to Gupta and Loh [31]. Furthermore, this study also showed that strain sensitivity was enhanced by patterning the films; the corresponding mechanisms that resulted in enhanced strain sensing performance were also investigated. These findings added new knowledge to the ECT and biomedical engineering fields, especially because early work only utilized ECT as an imaging tool for mapping cracks (or fractures) and voids [32]. The detailed imaging accuracy and resolution study also quantified ECT capabilities (i.e., based on the current hardware and algorithm implementations). The vision is that strain-sensitive nanocomposites will be integrated with pH-sensitive thin films (i.e., potentially as a multilayered structure) and deposited at the tissue-OIP interface for in situ and multi-modal sensing. This study was the first major step in realizing that vision.

## 7. ECT Imaging of Biological Specimens

**Figure 16:**
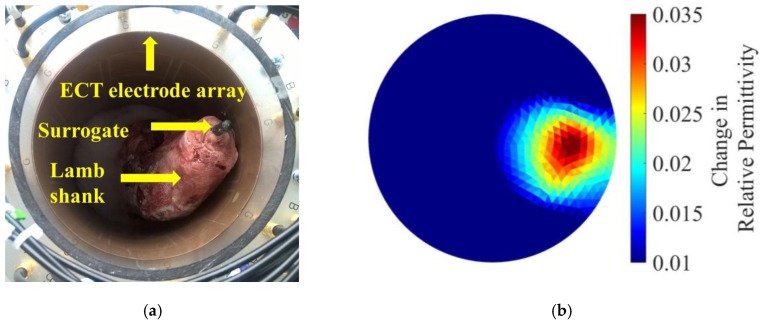
(**a**) The lamb shank with an embedded prosthesis phantom was scanned by the ECT electrode array. (**b**) The change in electrical permittivity distribution with respect to the empty baseline of air (i.e., empty ECT electrode array) is presented.

In order to further demonstrate the potential of ECT as an imaging tool for real biological tissues with high electrical conductivity, a simple experiment was carried out in the laboratory. A lamb shank was chosen as the test specimen, and a 6.35 mm-diameter steel rod, as an OIP surrogate, was press-fitted into the bone (Figure 16a). The lamb shank was then placed in the ECT electrode array for scanning, and the reconstructed permittivity distribution is shown in Figure 16b. It can be observed from Figure 16b that the shape of the lamb shank was successfully captured by ECT. A higher change in electrical permittivity was observed near the vicinity of the region where the prosthesis rod was located. A gradual decrease in permittivity is also seen radiating outward from the OIP region as a result of the presence of materials with lower dielectric properties (i.e., bone and soft tissue). Future tests will consider the embedment of a nanocomposite-coated prosthesis surrogate combined with load testing. These preliminary results demonstrate that ECT has the potential for use in biological applications. 

## 8. Conclusions

A noncontact, noninvasive, capacitance-based tomographic imaging technique was investigated for monitoring the strain and deformation states of osseointegrated prostheses. To achieve this goal, passive nanocomposite thin films, whose electrical permittivity changes in response to applied tensile and compressive strains, were designed as a coating for OIP implants. First, the ECT forward problem and a Gauss–Newton iterative inverse algorithm were implemented to reconstruct the permittivity of a predefined sensing region using only a limited number of noncontact boundary capacitance measurements. Second, the accuracy (based on three predefined metrics) of the proposed algorithm was investigated. After ensuring that the reconstructed images were of acceptable quality, ECT was employed for monitoring changes in electrical permittivity of various OIP phantom test specimens (i.e., the pristine specimen and specimens coated with two different thin films) subjected to two loading scenarios. It was found that ECT, when used alone and without a nanocomposite, was unable to detect strain-induced changes in electrical permittivity of the test specimens.

Thus, to overcome this limitation, a nanocomposite thin film whose permittivity is sensitive to strain was developed and characterized. It was found that patterning the film to form alternating strips of films and insulating coatings enhanced strain sensitivity. These films were then deposited onto OIP phantoms and subjected to both uniaxial and bending tests, while ECT was used to measure their strain-induced permittivity changes in a noncontact manner. Not only did the films enable noncontact strain monitoring, but it was also found that the patterned film exhibited ~4 times higher strain sensitivity. The patterned film could not only detect uniaxial strains, but bending tests also revealed that it could simultaneously monitor and localize tensile and compressive strains that developed on opposite faces of the test specimens. The results showed that permittivity varied linearly through the thickness of the specimen in a similar manner as one would expect for a cantilevered beam under bending. These results successfully validated noncontact strain sensing and open up new avenues for ECT to be used in conjunction with passive nanocomposite thin films for monitoring the strain-states of OIPs. Future research will focus on developing a wearable noncontact strain monitoring system and testing in more realistic environments.

## Figures and Tables

**Table 1:** Electrical capacitance tomography imaging accuracy evaluation results. *PE*—position error; *RES*—resolution; *SD*—shape deformation.

	Statistical Results	Mean	Standard Deviation
Parameters			
***PE* [mm]**		4.65 × 10^−4^	0.0017
***RES***		0.237	0.0133
***SD***		1.14 × 10^−4^	3.94 × 10^−5^
